# Recent advances in understanding Epstein-Barr virus

**DOI:** 10.12688/f1000research.10591.1

**Published:** 2017-03-29

**Authors:** Brent A. Stanfield, Micah A. Luftig

**Affiliations:** 1 Department of Molecular Genetics and Microbiology, Duke Center for Virology, Duke University Medical Center, Durham, NC, USA

**Keywords:** EBV, Herpesvirus, Herpes simplex virus, gastric cancer

## Abstract

Epstein-Barr virus (EBV) is a common human herpes virus known to infect the majority of the world population. Infection with EBV is often asymptomatic but can manifest in a range of pathologies from infectious mononucleosis to severe cancers of epithelial and lymphocytic origin. Indeed, in the past decade, EBV has been linked to nearly 10% of all gastric cancers. Furthermore, recent advances in high-throughput next-generation sequencing and the development of humanized mice, which effectively model EBV pathogenesis, have led to a wealth of knowledge pertaining to strain variation and host-pathogen interaction. This review highlights some recent advances in our understanding of EBV biology, focusing on new findings on the early events of infection, the role EBV plays in gastric cancer, new strain variation, and humanized mouse models of EBV infection.

## Introduction

Epstein-Barr virus (EBV), also known as human herpes virus 4, is a gamma-herpes virus that infects the majority of the world’s population. Initial infection with EBV is often asymptomatic but can also manifest as infectious mononucleosis. Following acute lytic replication in epithelial cells, EBV infects B cells where a distinct set of latency-associated genes and transcripts are expressed ^1^. EBV was first identified in 1964 from cultured tumor cells derived from a patient with Burkitt’s lymphoma (BL) ^2^. Early studies have demonstrated EBV’s ability to transform resting human B cells into lymphoblastoid cell lines (LCLs), further supporting the oncogenic potential of this virus ^3, 4^. Since then, EBV infection has been associated with a number of different malignancies of both lymphoid and epithelial origin and accounts for 1.8% of all cancer-related deaths worldwide ^5^.

*In vivo*, EBV infection begins in the oral mucosa. Replication in epithelial cells is typically lytic; however, latent infection of epithelial cells can result in nasopharyngeal carcinoma or gastric cancer (as discussed in more detail later). After replication in the epithelia, virus is primed for entry into B cells, where a transient growth program is thought to mimic a germinal center reaction, ultimately promoting maturation of the infected cell into the peripheral memory B-cell compartment. Advances in next-generation sequencing and the development of humanized mice have led to better ways to identify and understand the natural strain variation that occurs with EBV. New strain variations, particularly with mutations in latency-associated genes, have been identified in various malignancies. Harnessing these new humanized mice enables studies modeling latent infection and pathogenesis of host-restricted pathogens like HIV and EBV. This review will focus on the recent advances in EBV biology and primarily on understanding events in early B-cell infection, the role of EBV in gastric cancer, the breadth of EBV strain variation revealed by next-generation sequencing, and recent discoveries made using humanized mice.

## Early events

### Initial events of infection

**Figure 1.**
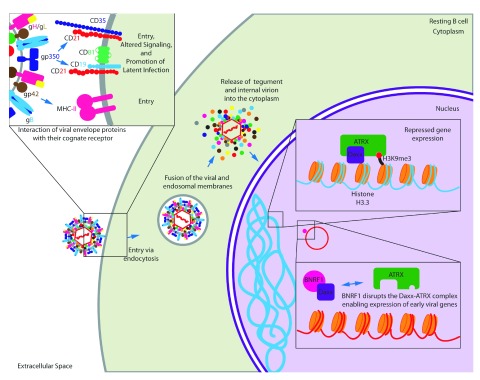
Initial events of Epstein-Barr virus (EBV) infection. The EBV membrane glycoprotein gp42 binds to its cell surface receptor major histocompatibility complex class II (MHC-II) to initiate entry into the cell. Also, gp350/220 binds to its cell surface receptor CD21 for entry. Interaction with CD21 initiates signaling cascades that prime resting B cells for persistent latent infection. Following endocytosis, the virion and packaged tegument proteins are released into the cytoplasm following fusion of the virion membrane with endosomal membrane. In particular, BNRF1 disrupts the Daxx/ATRX repressor complex to facilitate viral gene expression.

EBV entry into epithelial cells occurs by direct fusion of the viral envelope with the cell plasma membrane; however, entry into B cells requires the virus to be endocytosed before membrane fusion to escape the endosome ^6, 7^. B-cell entry requires five viral glycoproteins: gp350/220 allows for attachment by binding to CD21, gp42 binds to major histocompatibility complex (MHC) class II to initiate entry ^8^, and the core herpes-virus fusion machinery consisting of gB and the heterodimer gH/gL ( Figure 1) ^1^. Interaction of gp350/220 with the cell surface molecule CD21 results in the alteration of major signaling pathways believed to prime the cell to stable latent EBV infection. In particular, specific transcriptional profiles are involved in the evasion of apoptosis and there is evidence that EBV/CD21 binding alters the expression of specific histone transcripts from clusters 1 and 2 (H2AFC, H2AFM, H2BF, H2BFG, H2BI, H3FA, H3FB, H3FL, H4FL, H4FK, H4FI, H4FK, H4F2, H1F3, and H1F4) ( Figure 1) ^9^.

Upon entry into B cells, the virion is endocytosed and is released into the cytoplasm following fusion of the virion membrane with endosomal membrane. This process releases the viral tegument proteins into the host cell. One such tegument protein, BNRF1, binds the cellular protein Daxx and disrupts the Daxx-ATRX complex ^10^. This complex is known to suppress transcription through histone methylation ^11^. Upon deposition into the nucleus, the viral DNA is associated with cellular histones ^12^. Daxx-ATRX might normally support methylation of this new EBV chromatin to suppress transcription of viral genes. However, BNRF1 disruption of the Daxx-ATRX complex allows early viral latent gene expression ( Figure 1) ^10^.

Packaged, virally encoded RNA is also released upon fusion of the virion membrane with endosomal membrane. In particular, BZLF1 transcripts have been shown to be packaged into viral particles and are translated immediately upon release into the infected cell. These immediately translated proteins then function to transactivate viral promoters initiating the pre-latent phase of EBV infection. EBV also counters T-cell responses through the delivery of BNLF2a mRNA and non-coding EBV-encoded RNA transcripts that induce cellular cytokine synthesis ^13^. EBV is known to encode at least 44 microRNAs (miRNAs). Though many of the miRNAs have no known function, it has recently been shown that these virally encoded miRNAs function in immune evasion by specifically suppressing the release of interleukin-12 (IL-12), disrupting CD4 ^+^ T-cell differentiation into type 1 T helper (Th1) cells, and reducing antigen presentation to CD4 ^+^ and CD8 ^+^ T cells. These miRNAs function by interfering with peptide processing, by directly targeting the TAP2 subunit, and by disrupting antigen presentation on MHC-II and MHC-I molecules ^14, 15^.

### Pre-latent gene expression

Pre-latent gene expression occurs immediately upon deposition of the viral genome into the nucleus of newly infected B cells. Promiscuous expression of both lytic and latent genes occurs at this time with the majority of infected B cells initially expressing EBV immediate-early genes ^16^. Others have shown that BZLF1, the major transcriptional activator of lytic gene expression, is expressed as early as 1.5 hours post infection in the absence of protein expression, implicating BZLF1 as an immediate-early gene being expressed immediately following B-cell infection ^17^. This initial burst of lytic gene expression could be essential to the production of progeny virus competent for infecting new B cells ^18^, or immediate expression of lytic genes could be essential for the survival of latently infected B cells through inactivation of p53 ^19^. However, it is important to note that during this pre-latent phase, genes essential for DNA replication and structural proteins of the virion are not readily detectable ^16^.

EBV encodes two bcl-2 proteins: BHRF1 and BALF1. Viral mutants lacking both bcl-2 proteins are unable to initiate proliferation and die from immediate apoptosis. Peak expression of these transcripts is detected at 24 hours post infection, implicating BHRF1 and BALF1 in the initial events prior to cell proliferation ^20^. Also, it has recently been shown that BHRF1 is constitutively expressed as a latent protein in BamHI W promoter (Wp)-restricted BL cell lines and LCLs ^21^. These findings implicate BHRF1 and BALF1 proteins as playing an important role in the evasion of apoptosis during latency; however, virally encoded miRNAs also cluster at the *BHRF1* locus. Following induction of viral replication in latency I restricted Akata cells, these miRNAs are detectable at 24 hours post stimulation and have been shown to drive proliferation and aid in the evasion of apoptosis ^22– 24^.

### Hyper-proliferation

Following the pre-latent phase, the initial Epstein-Barr nuclear antigen (EBNA) latency promoter, Wp, is active promoting expression of *EBNA-LP* and *EBNA2*. Subsequently, these proteins transactivate the viral C promoter, Cp, to initiate expression of the EBNA3s and EBNA1 along with their own transcripts to high levels. This EBNA-only gene expression state is associated with a period of rapid proliferation with the first three or four divisions occurring once every 8 to 12 hours ^25^. This period lasting approximately the first 2 weeks following resting B-cell infection is termed latency IIb ^26, 27^. At this time, the virus expresses all of the EBNA proteins and minimally expresses latent membrane proteins (LMPs) 1, 2A, and 2B ^26^. LMP1 is expressed as early as 2 days post infection; however, during this period, inhibition of early nuclear factor-kappa B (NFκB) activation does not affect transformation, supporting the distinction of this latency phase from the LCL state, which requires LMP1-mediated NFκB activity for survival ^26^.

As a consequence of this rapid proliferation, EBV-infected B cells are susceptible to growth arrest induced by hyper-proliferation-associated DNA damage response ^26, 28^. Cells then transition from a period of rapid proliferation and high Myc activity to the steady-state proliferation (about 24 hours per cycle) observed in LCLs with lower Myc and high NFκB activity ^26, 29, 30^. The high Myc/low NFκB state that occurs during latency IIb might play a role in immune evasion as elevated Myc and low NFκB as observed in BL have been implicated in downregulation of MHC class I and II (MHC-I and MHC-II) and avoidance of T-cell recognition and killing ^29, 30^.

## Epstein-Barr virus infection in gastric cancer

Viral entry into epithelial cells is primarily mediated by three CD21-independent mechanisms. First, EBV can enter into epithelial cells by close membrane-to-membrane contact of EBV-infected lymphocytes to uninfected epithelial cells. Second, cell-free virus can enter polarized epithelial cells through their basolateral membranes which is mediated in part by interaction between BMRF2 and beta1 and alpha5beta1 integrins. The third mechanism is by lateral spread through the epithelium from infected to uninfected epithelial cells ^31^. EBV-associated gastric carcinomas (EBVaGCs) are epithelial in origin and make up approximately 9% of all gastric carcinomas worldwide ^32^. EBVaGCs characteristically acquire mutations within the cellular *PIK3CA* gene and display extreme cellular DNA hyper-methylation. Specifically, mutations in *PIK3CA* identified in intestinal-type gastric cancers were associated with an increased tumor incidence in the lower third of the stomach compared with those without ^33^. Also, *PIK3CA* mutations in diffuse-type gastric cancer were associated with an increased tumor incidence in the upper third of the stomach and an increased association with hematogenous metastasis. Tumors identified with *PIK3CA* mutation in the middle third of the stomach had an increased association with EBV infection and increased peritoneal recurrence; however, *PIK3CA* mutations did not demonstrate a significant effect on patient outcomes.

EBVaGCs are also known to have increased expression of JAK2, programmed death ligand 1 (PD-L1), and PD-L2 ^34^. PD-L1 is known to interact with programmed death receptor 1 found on the surface of T cells. This interaction causes the inhibition of T-cell proliferation, cytokine secretion, and cytotoxic activity (reviewed in 35). Also, EBVaGCs have been shown to express BNLF2a, which functions in immune evasion by inhibiting the transporter associated with antigen-processing transport of antigenic peptides. Though this transcript is typically associated with lytic replication, in gastric cancers it is expressed latently and has the potential to protect the infected cell from immunosurveillance ^36^. Despite the immunologically evasive nature of EBVaGC, patients with diagnosed EBVaGC had longer survival post diagnosis as opposed to EBV-negative gastric carcinoma ^37^.

Infection of an EBV-negative GC cell line (AGS) with Akata EBV results in robust expression of virally encoded BART miRNAs with minimal protein expression ^38^. Importantly, these infected AGS cells displayed a more transformed phenotype than their uninfected counterparts. The prototypical transforming EBV strain, B95-8, readily infects and immortalizes human B cells. However, this virus is deleted for most of the BART miRNAs, and infection of B cells with viral variants encoding these miRNAs results in minimal BART expression ^39, 40^. This tissue-specific BART expression suggests that these miRNAs are likely to play a significant role in the transformed growth properties of EBVaGC.

Recently, it has been shown that CRISPR/Cas9-mediated cleavage for bacterial artificial chromosome (BAC) insertion into EBV episomal DNA in gastric carcinoma (GC) cell lines has facilitated the cloning of these viral genomes with unprecedented efficiency ^41^. Subsequent infection of epithelial cells with the BAC clone reconstituted viruses induced resistance to oncogene-induced cell death, providing important clues concerning EBV-mediated epithelial carcinogenesis. Establishing this new state-of-the-art technique will enable future investigation into new strain variations and their relationship with EBV-associated disease.

## Epstein-Barr virus strain variation

Recent advances in next-generation whole genome sequencing (NGS) have changed the landscape surrounding the analysis of EBV-type differences. Historically, the major distinction in EBV strains has been the delineation of type 1/type 2. Currently, the largest distinguishing factors between EBV type 1 and type 2 rely on differences observed in the *EBNA2* and *EBNA3A*, *EBNA3B*, and *EBNA3C* genes. Indeed, it has been shown that a single amino acid change in the transactivation domain of EBV-2 EBNA2 (S442D) can drastically alter EBV-2 B-cell transformation efficacy similar to that observed with EBV-1 and increase induction of LMP1 expression with a higher affinity for the LMP1 promoter ^42^. However, a number of other factors may contribute to the underlying strain variation, including immunological pressures, skewed cell tropism, and geographic isolation ^43^. Indeed, a recently described strain of EBV derived from a nasopharyngeal carcinoma case, M81, displays high epithelial tropism and also contains a polymorphism in the promoter of the lytic transactivator BZLF1 leading to elevated lytic replication ^44^.

It has been proposed that the prevalence of MHC haplotypes within specific geographic regions induces immunological pressures that can contribute to strain variation within immunologically dominant epitopes of particularly immunogenic proteins ^45^. However, recent sequence analyses demonstrate that the large numbers of non-synonymous mutations observed in the EBNA3 proteins are outside of known cytotoxic T-cell epitopes. More work is needed to identify alternative cytotoxic T lymphocyte (CTL) epitopes within the EBNA3s to explain this variation, or alternatively another selective pressure could be driving this variation perhaps regarding EBNA3 function ^43^. For example, a recent study found that EBNA3B, an immunodominant latency protein, actually serves as a tumor suppressor and can be found deleted in EBV strains associated with diffuse large B-cell lymphomas (DLBCLs) ^46^.

Recently, a provocative study implicated EBV-2 as having unique cell tropism skewing toward CD8 ^+^ T cells ^47^. EBV has also been commonly detected in non-B cells in the blood of patients with EBV-positive lymphoproliferative disorder (LPD), including patients with HIV, post-transplant, anaplastic anemia, chronic active EBV (CA-EBV), and others ^48, 49^. While CA-EBV patients often had EBV+ T cells in the blood, other EBV+ LPD patients contained EBV in monocytes as well as non-B, non-T, non-monocyte cell types based on surface staining ^49^. Although this population is certainly skewed from the norm with elevated viral loads and altered EBV immune responses, these findings suggest that EBV infection of T cells may be clinically relevant in some instances. Indeed, the detection of EBV in natural killer (NK)/T lymphomas ^50^ and a high percentage of T cells in EBV-associated hemophagocytic lymphohistiocytosis (HLH) ^51^ suggest that lack of control of EBV infection might be associated with a broadening of cellular tropism. Interestingly, cases of CA-EBV are most commonly reported as being of T and NK cell origin in Asia ^52^ and almost entirely B-cell origin in the United States ^53^. Information gained through NGS studies coupled with further virus-host interaction work *in vitro* and clinical observation will lead to a greater understanding of how different EBV strains might achieve these drastic differences in cellular tropism and maintenance of latency in various cell types.

## Humanized mouse models of Epstein-Barr virus infection

EBV infection had been restricted to *in vitro* systems until the breakthrough of the scid-hu PBL mouse. Scid-hu PBL mice are based on the C.B-17 severe combined immunodeficient (SCID) mouse, which lack both B and T cells ^54^. These mice are injected with human peripheral blood mononuclear cells and, after infection with EBV, effectively model the LPD observed in immunocompromised humans (reviewed in 55). However, these mice have several drawbacks, including frequently observed graft-versus-host disease caused by the human T cells attacking mouse tissue, the transient nature of the engrafted human immune system, and a relatively low level of engraftment. Most importantly, these mice are unable to mount adaptive immune responses with their engrafted immune systems.

In order to overcome the obstacles of the scid-hu PBL mouse model, a new suite of humanized mice was generated by transplantation of non-obese diabetic/SCID (NOD/SCID) animals with hematopoietic stem cells. These NOD/SCID mice have a complete null mutation of the common IL-2 cytokine receptor gamma chain—NOD/LtSz-scid/ *IL-2* receptor gamma null (NSG), NOD/Shi-scid/ *IL-2* receptor gamma null (NOG)—and, once transplanted, display a humanized immune system that persists for more than 24 weeks post transplant (reviewed in 56). In this model, the CD34 ^+^ hematopoietic stem cells are able to differentiate into various mature blood cells, including myelomonocytes, dendritic cells, erythrocytes, platelets, and lymphocytes. B cells undergo normal class switching, produce normal immunoglobulins, and even infiltrate into mucosal tissues in these mice. However, it is important to note that circulating IgG is approximately 1,000 times lower than that observed in immunocompetent humans and that infiltration into mucosal tissues has been demonstrated to be severely attenuated. Differentiated T cells display human MHC-I/HLA-restricted cytotoxic functions: a vast improvement over scid-hu PBL mice ^57^. The introduction of the human *HLA A2* allele into NSG mice transplanted with CD34 ^+^ hematopoietic stem cells (NSG- *HLA-A2*) resulted in mice capable of reproducing adaptive immune responses known to occur after EBV infection of HLA A2-expressing individuals ^58^. These NSG- *HLA-A2* mice have been used to demonstrate the essential contribution of NK cells in controlling EBV infection with NK depletion resulting in the development of disseminated EBV ^+^ lymphomas ^59^. Further still, the BLT-NOD mice were developed by transplantation of autologous human hematopoietic fetal liver CD34 ^+^ cells into NOD/SCID mice previously implanted with human fetal thymic and liver tissues. This resulted in long-term, systemic human T-cell homeostasis capable of mounting anti-EBV MHC-I and MHC-II restricted adaptive immune responses ^60^. Given the vast improvements in small animal models of EBV infection, we now have the tools to study post-transplant LPD in the context of a human immune system, adaptive immune responses to EBV infection, and an experimental model to understand the *in vivo* effects of strain variation and other important biological questions.

Humanized mice have been shown to demonstrate the cardinal features of EBV-associated diseases developing B-cell LPD, EBV-associated HLH, and erosive arthritis resembling rheumatoid arthritis (RA). NOG humanized mice injected with 10 ^3^ 50% transforming dose (TD50) of EBV develop B-cell LPD. This LPD models the histological and viral gene expression signature observed in immunocompromised patients. Lower dose infection of less than or equal to 10 TD50 in NOG humanized mice resulted in a persistent asymptomatic infection with adaptive CD8 ^+^ T-cell responses and virus-specific IgM detectable in the serum of infected animals ^61^. Infection of NOG humanized mice has also been shown to result in the cardinal features of HLH with infected animals developing hemophagocytosis, erythrocytopenia, thrombocytopenia, hypercytokinemia, histiocyte proliferation and infiltration of activated CD8 ^+^ T cells into the spleen ^62^. EBV has been implicated in the pathogenic manifestation of RA. Patients with this disorder demonstrate elevated EBV reactive antibody titers and impaired lymphocyte responses to EBV, and EBV has been identified in the synovial fluid of patients with RA, indirectly implicating EBV in RA pathogenesis ^63– 65^. Modeling this pathological phenotype, humanized NOG mice infected with EBV develop an erosive arthritis. However, these findings are purely morphological and require in-depth molecular characterization to further validate this model ^66^. A detailed description of recent publications involving the use of humanized mice in EBV research can be found in Table 1.

**Table 1.** Epstein-Barr virus humanized mouse studies.

Mouse	Epstein-Barr virus strain	Year	Findings	References
NSG+CD34-depleted human cord blood mononuclear cells	M81 BAC and p2089 B95-8 LMP1-KO	2016	Blocking PD-1/CTLA-4 inhibits Epstein-Barr virus (EBV)-induced lymphoma growth.	67
NSG+purified CD34-positive cells from individual fetal liver samples	GFP-EBV B95-8 WT	2016	Leukocytes lacking cognate HLA ligands interfere with KIR ^+^ natural killer (NK) recognition of HLA- tumors but improve NK-mediated control of EBV infection.	68
NSG-A2tg (expressing HLA *-A2*)+purified CD34-positive cells from two fetal liver samples	M81BAC, M81BACΔC1, M81BACΔC2, M81BACΔC1C2, M81BACΔb2, and M81BACΔAll	2015	BART microRNAs repress tumorigenesis *in vivo* and likely facilitate long-term persistence in the infected host.	69
Rag2 ^−/−^ γC ^−/−^ double knockout+human hematopoietic stem cells injected into the liver	293EBV ^+^ and 293EBVdelta (BPLF1-KO)	2015	BPLF1 contributes to EBV oncogenicity.	70
NSG+purified human cord blood CD34-positive hematopoietic stem cells injected into the liver	B95-8	2015	EBV-associated Hodgkin’s lymphoma develops exclusively in mice with activated T-cell conditions and EBV-associated non-Hodgkin’s lymphoma develops in mice with a largely suppressed T-cell condition predominantly characterized with an abundance of immature B cells.	71
NSG-A2tg +purified human cord blood CD34-positive hematopoietic stem cells injected into the liver	B95-8 BAC, EBER1 or EBER2 deletion mutants, and revertant viruses	2015	Wild-type and EBER-deleted mutant viruses demonstrate equal ability to persist *in vivo*.	72
NSG+purified human fetal liver CD34-positive hematopoietic stem cells injected into the liver	B95-8 *GFP* ^+^	2015	The human SAP-dependent 2B4 receptor is required for CD8 ^+^ T cell-mediated control of EBV infection.	73
NSG+purified CD34-positive cells from individual fetal liver samples and fetal thymus from the same donor	p2089 B95-8 BAC and p2089 B95-8 BAC LMP1-KO	2015	LMP1 is not essential for EBV-induced lymphomas *in vivo*, and T cells supply signals that substitute for LMP1 in EBV-positive B-cell lymphomagenesis.	74
NSG-A2tg +purified human cord blood CD34-positive hematopoietic stem cells injected into the liver	Wild-type B95-8 and BZLF1 knockout	2014	T cells specific for the lytic EBV antigen BMLF1 can effectively control lytically replicating EBV ^+^ B cells *in vivo*.	75
Rag2 ^−/−^ γC ^−/−^ double knockout+human peripheral blood mononuclear cells (PBMCs) or Vγ9Vδ2-T cell- depleted PBMCs	B95-8 and B95.8EBfaV- *GFP*	2014	Vγ9Vδ2-T cells contribute to EBV immunity.	76
NSG+purified CD34-positive human cord blood mononuclear cells	B95-8	2014	CD4 ^+^ T cells are necessary for the generation/ maintenance of cells with latency I/IIa phenotype in humanized mice and contribute to this process through expression of CD40L.	77

## Concluding remarks

The recent advances described in this review address many of the key questions facing the EBV field today. With the advent of NGS and the development of humanized mice to better model EBV disease *in vivo*, we now have the tools to better understand the effects of strain variation on the development of EBV-associated diseases. Future research will benefit from further refinement of the humanized mouse models to better model the full spectrum of the human immune response to EBV infection with the aim of developing effective EBV-specific prophylactics and therapeutics. Further studies of the early period after B-cell infection and its contribution to tumorigenesis and immune evasion will be important to study in the humanized mouse. Finally, the role of EBV in epithelial malignancies and other diseases outside of the B-cell compartment is ripe for study in this post-genomic era of EBV biology.
